# Synthesis, electronic properties and self-assembly on Au{111} of thiolated (oligo)phenothiazines

**DOI:** 10.3762/bjoc.6.72

**Published:** 2010-07-02

**Authors:** Adam W Franz, Svetlana Stoycheva, Michael Himmelhaus, Thomas J J Müller

**Affiliations:** 1 Institut für Organische Chemie und Makromolekulare Chemie, Heinrich-Heine-Universität Düsseldorf, Universitätsstr. 1, D-40225 Düsseldorf, Germany; 2 Physikalisch-Chemisches Institut, Ruprecht-Karls-Universität Heidelberg, Im Neuenheimer Feld 253, D-69120 Heidelberg, Germany

**Keywords:** cyclic voltammetry, ellipsometry, phenothiazines, SAM, thiols

## Abstract

(Oligo)phenothiazinyl thioacetates, synthesized by a one-pot sequence, are electrochemically oxidizable and highly fluorescent. SAMs can be readily formed from thiols prepared by in situ deprotection of the thioacetates in the presence of a gold-coated silicon wafer. Monolayer formation is confirmed by ellipsometry and the results compared to those obtained by force field and DFT calculations.

## Introduction

Functional organic π-systems [1] are of great relevance in the miniaturization of electronic devices particularly since they could serve as molecular switches, wires, and transistors [2–5]. As a consequence, the molecule-based bottom-up approach to nanodimensional structured self-assembled monolayers (SAMs) on well-defined metal surfaces has become a groundbreaking strategy in the development of molecular electronics [6]. In recent years, many investigations into SAMs of organic molecules on gold surfaces have been carried out [7]. Thiols, thiol esters, and disulfides can be easily chemisorbed on gold to form SAMs by exposure of well-defined gold substrates to solutions of sulfur functionalized molecules [7–14]. These “alligator-clips” [15–17] are able to bind functional molecules covalently to gold{111}-surfaces. Phenyl derivatives [18–19], conjugated bi- [18,20] and oligophenyls [18,20–21], oligothiophenes [18], porphyrin derivatives [20], phenanthrenes [22–23], fullerenes [24], and optically active naphthalenes [25] adsorbed on gold were studied in break-junction experiments and their properties on conductance, 1-bit random access memory and, especially, with regard to their ability to function as conductive molecular wires investigated. Among many heteroaromatic systems, phenothiazines, their derivatives and oligomers are interesting building blocks for rigid-rod and wire-like molecular modules for single-molecule electronics as a consequence of their electronic properties. In particular, their reversible formation of stable radical cations [26–31], their tunable redox and fluorescence properties [32–34], and their tendency to self-assemble on surfaces by π–π interactions [35] make them eligible for use as redox-switchable molecular entities. In addition, the inherent folded conformation of phenothiazines [36], with a folding angle of 158.5°, represents an intriguing new aspect for the formation of self-assembled monolayers (SAMs) of this class of compounds. Furthermore, the transformation of phenothiazines into stable planar radical cations with excellent delocalization [37] qualifies them as excellent models for switchable conductive or semiconductive molecular wires. Encouraged by successful electrode modifications with conjugated thiolated anilines [38] and SAM formation of thiolated phenylethynyl phenothiazines [39], and in continuation of our investigations directed towards the synthesis and study of (oligo)phenothiazine-based functional π-systems [40–46], we have now focused our attention on thiolated phenothiazines and (oligo)phenothiazines as “alligator-clips”. Here, we report the synthesis of phenothiazines and their oligomers bearing “alligator-clips” and their electronic properties as studied by cyclic voltammetry (CV), spectroscopic and spectrometric methods. Furthermore, their chemisorption and SAM formation on Au{111} were studied by ellipsometry.

## Results and Discussion

### Synthesis

**Scheme 1:**
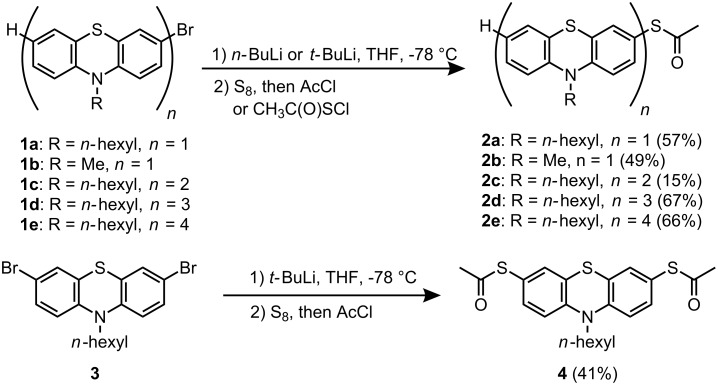
Synthesis of (oligo)phenothiazinyl thioacetates **2** and **4**.

The facile bromine–lithium exchange of bromo phenothiazines [47–49] and the subsequent electrophilic trapping reactions of the resulting lithio phenothiazines [50–51] with different electrophiles set the stage for a straightforward synthesis of thiolated (oligo)phenothiazines. Therefore, the synthesis of thiofunctionalized phenothiazines can be accomplished according to a standard protocol [18]. Thus, solutions of bromo phenothiazines **1** [32,52] were cooled to −78 °C and reacted with *n*-BuLi (**1a** and **1b**) or *t*-BuLi (**1c–e** and **3**), respectively, to give the corresponding lithio phenothiazines via bromine–lithium exchange. Subsequent addition of elemental sulfur, followed by stirring for 5 min at −78 °C, and the addition of freshly distilled acetyl chloride furnished the desired (oligo)phenothiazinyl thioacetates **2** and **4** in moderate to good yields (Scheme 1). However, in the case of dyad **1c** thiolation was only accomplished by addition of acetylsulfur chloride [53] to the lithio species at low temperature, albeit the thiofunctionalized derivative **2c** was obtained in only 15% yield. The structures of the (oligo)phenothiazinyl thioacetates **2** and **4** were unambiguously supported by ^1^H and ^13^C NMR spectroscopy, mass spectrometry and elemental analysis.

### Electronic properties

**Figure 1:**
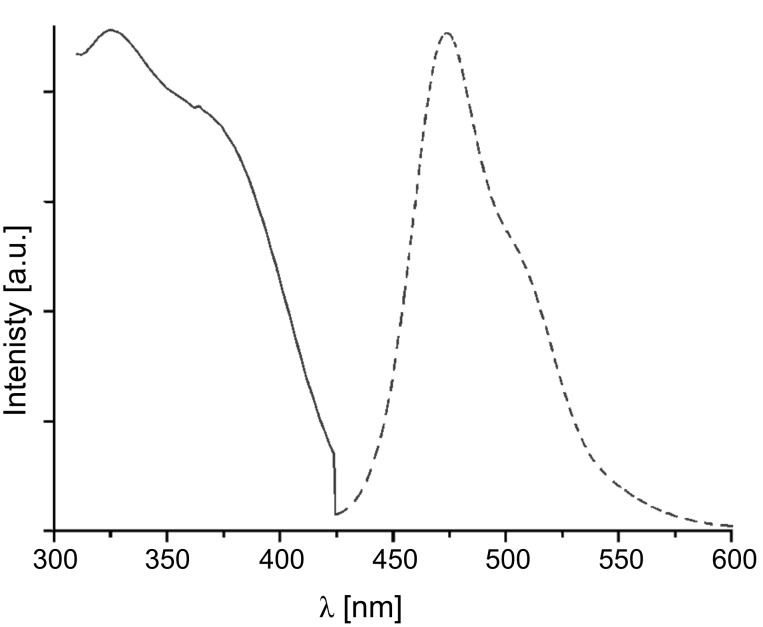
Normalized absorption (solid line) and emission (dashed line) spectra of thioacetate **2d** (recorded in dichloromethane, *T* = 298 K).

The electronic properties of the (oligo)phenothiazinyl thioacetates **2** and **4** were investigated by absorption and emission spectra, and cyclic voltammetry (Table 1). Optical spectroscopy (UV–vis and fluorescence spectra) revealed that only the triad **2d** and the tetrad **2e** displayed considerable fluorescence with emission of greenish-blue light and large Stokes shifts (Figure 1, Δ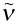 6400–6600 cm^−1^). While the absence of fluorescence of monophenothiazines **2a**, **2b**, and **4** with heavy atom substitution and consequently, increased spin–orbit coupling is not too surprising, the presence of a diphenothiazine unit (**2c**) is not sufficient. Hence, at least two covalently bound phenothiazines without an additional sulfur substituent appears to be the prerequisite for intense fluorescence of oligophenothiazinyl thioacetates.

**Table 1:** Selected electronic properties of (oligo)phenothiazinyl thioacetates **2** and **4** (absorption^a^ and emission spectra^a^ and cyclic voltammetry^b^).

	Absorption *λ*_max,abs_ (nm)	Emission *λ*_max,em_ (nm)	Stokes shift Δ 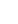 (cm^−1^)	*E*_0_^0/+1^ (mV)	*E*_0_^+1/+2^ (mV)	*E*_0_^+2/+3^ (mV)

**2a**	266, 310	–	–	800	–	–
**2b**	264, 316	–	–	838	–	–
**2c**	276, 324, 366	–	–	668	853	–
**2d**	280, 326, 364	474	6400	608	765	876
**2e**	282, 326, 362	476	6600	597	690	842^c^
**4**	272, 326	–	–	875	–	–

^a^Recorded in CH_2_Cl_2_. ^b^Recorded in CH_2_Cl_2_, 20 °C, *v* = 100 mV/s, electrolyte: *n*-Bu_4_N^+^PF_6_^−^, Pt working electrode, Pt counter-electrode, Ag/AgCl reference electrode. ^c^The third and fourth oxidation waves coincide.

**Figure 2:**
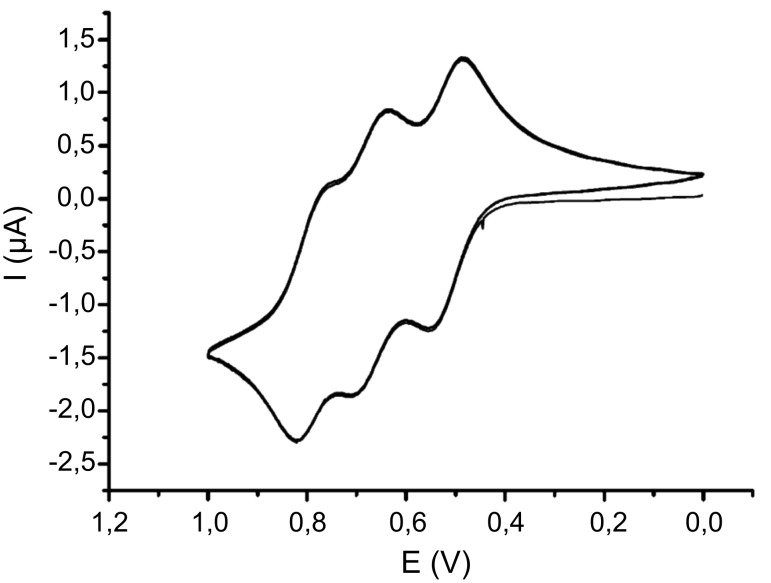
Cyclic voltammogram of thioacetate **2d** (recorded in CH_2_Cl_2_, *T* = 293 K; 0.1 *M* electrolyte [Bu_4_N][PF_6_]; *ν* = 100 mV/s; Pt-working electrode, Ag/AgCl-reference and Pt-counter electrode.

Electrochemical data for (oligo)phenothiazinyl thioacetates **2** and **4** were obtained by cyclic voltammetry in the anodic region (scan area up to 1.5 V). The reversible first oxidations to the radical cations of monophenothiazines **2a**, **2b**, and **4** were shifted anodically in comparison to unsubstituted monophenothiazines [54] as a consequence of the electron-withdrawing nature of the thioacetate. Due to unsymmetrical substitution, the dyad **2c** showed two distinctly separated, reversible oxidations at *E*_0_^0/+1^ = 668 mV and *E*_0_^+1/+2^ = 853 mV. The cyclic voltammogram of the triad **2d** displayed three distinctly separated, reversible oxidations at *E*_0_^0/+1^ = 608 mV, *E*_0_^+1/+2^ = 765 mV, and *E*_0_^+2/+3^ = 876 mV (Figure 2). However, the electrochemistry of the tetrad **2e** is more complicated. Only three distinctly separated, reversible oxidations were evident. The first oxidations at *E*_0_^0/+1^ = 597 mV and *E*_0_^+1/+2^ = 690 mV are in accordance with Nernstian behavior, while the third oxidation at *E*_0_ = 842 mV reveals a large difference of Δ*E* = 132 mV for the current peaks of the oxidation and the reduction wave. Presumably, the expected third and fourth oxidations coincide and give rise to a combined quasi-reversible peak.

### Self-assembly and ellipsometry

**Scheme 2:**
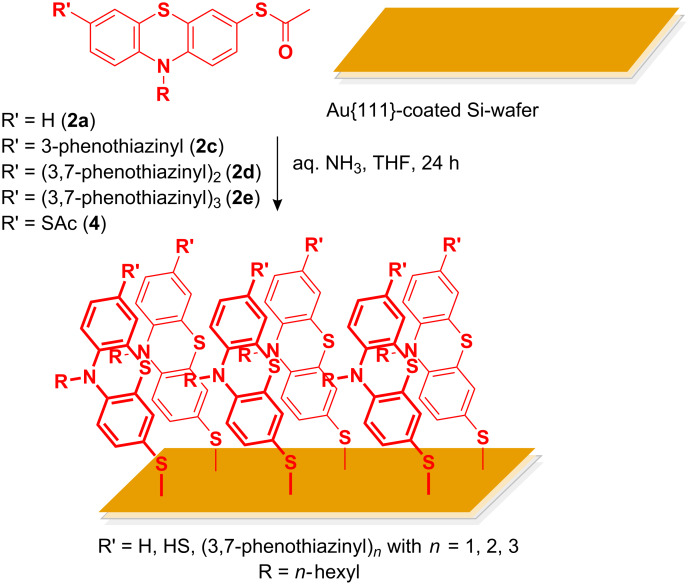
Preparation of SAMs from (oligo)phenothiazinyl thioacetates **2** or **4** on a Au{111}-coated silicon wafer substrate.

SAMs on a Au{111}-coated silicon wafer substrate were prepared from (oligo)phenothiazinyl thioacetates **2** or **4** by in situ saponification with degassed aqueous ammonia in THF at room temperature for 24 h (Scheme 2).

Based upon thorough surface analysis of the previously studied thiolated phenylethynyl phenothiazines chemisorbed on Au{111} by ellipsometry, contact angle measurements, X-ray photoelectron spectroscopy, and infrared reflection absorption spectroscopy (IRRAS) [39], we applied ellipsometry in combination with molecular modeling at the force field and DFT levels of theory for the characterization of SAMs of in situ liberated (oligo)phenothiazinyl thiols on Au{111}. The ability of the molecules to form SAMs was investigated by solution adsorption of different systems onto gold films of 100 nm thickness thermally evaporated onto Si wafers using 10 nm of Ti as adhesion promoter. This procedure is known to yield polycrystalline gold films with preferential {111} orientation [55].

The thickness of the layer was determined by ellipsometry as described above. As an estimate for the molecular dimensions of the monolayers, the structures of the (oligo)phenothiazines **2** and **4** were computed at the MM2 and DFT levels of theory (Table 2) [56].

**Table 2:** Measured (ellipsometry) and calculated (MM2, DFT) layer thickness of (oligo)phenothiazinyl thioacetates **2a**, **2c–e**, and **4** on Au{111}-coated silicon wafers.

Compound^a^	Measured layer thickness *d*_exp_^b^ (Å)	Calculated molecule length *l*_mol_		Calculated layer thickness *d*_th_^d^		Coverage *θ*^e^		Monolayer
		MM2 (Å)	DFT^c^ (Å)	MM2 (Å)	DFT^c^ (Å)	MM2 (%)	DFT^c^ (%)	

**2a**	9.0 ± 1.00	9.04	9.10	10.4	10.5	86.6 ± 9.56	86.1 ± 9.50	Yes
**2c**	11.4 ± 0.99	17.5	15.3	18.2	16.2	62.6 ± 5.41	70.4 ± 6.09	Poor
**2d**	18.0 ± 1.44	19.1	21.6	19.7	22.0	91.5 ± 7.32	81.9 ± 6.56	Yes
**2e**	10.9 ± 1.61	25.5	22.4	25.6	22.7	42.6 ± 6.28	48.0 ± 7.07	Poor
**4**	12.9 ± 1.06	11.7	10.4	12.9	11.7	100.2 ± 8.22	110.5 ± 9.07	Yes

^a^Thioacetate precursor. ^b^Measured by ellipsometry. Errors given are the figures of merit of the least squares fitting routine as determined by the ellipsometer built-in software. ^c^DFT calculations (B3LYP/3-21G), the hexyl group was replaced by a methyl group [28]. ^d^*d*_th_ = *l*_mol_ cos *φ* + *l*_Au-S_; *l*_Au-S_ = 2.1 Å; *φ*_anthracene-2-thiol_ = 23°. ^e^*θ* = *d*_exp_/*d*_th_.

To minimize computational time in the latter calculations, the hexyl substituents were truncated to methyl groups. From these calculations, the theoretical layer thickness was calculated according to *d*_th_ = *l*_mol_ cos φ + *l*_Au–S_, where *l*_mol_ is the calculated length of the respective molecule, φ is the molecules’ tilt angle with the surface normal, and *l*_Au–S_ = 2.1 Å is the Au–S bond length [57]. For φ, we refer to a recent electron spectroscopic analysis on similar aromatic systems, which determined φ = 23° for anthracene-2-thiol [58]. Using this value, we made the reasonable assumption that the Au–S–C bond is mainly influenced by the adjacent phenyl system. Table 2 shows *d*_th_ for the different molecules along with the experimental thickness *d*_exp_ as determined by ellipsometry. The theoretical thicknesses are given for MM2 as well as DFT calculations. As a simple measure of monolayer formation of the different systems, the relative coverage θ obtained experimentally is calculated from θ = *d*_exp_/*d*_th_ as given in Table 2. From these values it is clear that of **2**, only **2a** and **2d** show good SAM formation, suggesting an odd–even effect on film growth, which might be related to steric hindrance during adsorption when an even number of phenothiazine units are present, e.g., because of a back bending of the thiol-bound molecule to the gold surface in these cases, supported by additional gold-π-interactions with the terminal phenothiazine, which thus would hamper the formation of a SAM with an almost parallel intermolecular orientation. In corroboration of such disorder effects, coverage seems to decrease with increasing molecule length for even-numbered molecules (cf. Table 2). The highest coverage was obtained with **4**, which is not surprising, because the thiol bifunctionality allows chemisorption of the molecule at either side, which reduces the impact of steric effects on the adsorption kinetics and thus may lead to a more densely packed film. As a consequence, thiolated mono- and terphenothiazines **2** (*n* = 1, 3) and the dithiolated derivative **4** can be easily self-assembled to give stable monolayers on gold surfaces. This feature makes this class of redox-active molecular entities highly interesting for the fabrication of functionalized electroactive surfaces and nanostructured devices.

## Conclusion

In summary we have shown a concise, general synthetic access to (oligo)phenothiazinyl thioacetates that are suitable precursors for the formation of thiol-bound (oligo)phenothiazines on gold surfaces. Whereas the first oligomers are non-fluorescent, the triad and the tetrad display intense greenish-blue fluorescence in addition to distinct multiple reversible oxidation. The in situ deprotection of the thioacetates to thiols in the presence of a gold-coated silicon wafer was used to prepare self-assembled monolayers, which were unambiguously characterized by ellipsometry and accompanying force field and DFT calculations. The chemical trigger of gradual thiol liberation enables better control of film formation and adsorption kinetics, which can be very useful, for example, for co-adsorption of the moieties with a second, nonconductive molecule, which serves as an insulating matrix. Further studies directed toward such more-complex (oligo)phenothiazine SAMs on gold and functionalized redox manipulable surfaces, the nanoscopic characterization of the monolayers as well as their manipulation with external stimuli are currently underway.

## Experimental

### General considerations

Reagents, catalysts, ligands, and solvents were purchased reagent grade materials and used without further purification. THF and acetyl chloride were dried and distilled according to standard procedures [59]. The bromo phenothiazines **1a** [50–51], **1b** [50–51], **1c** and **1d** [32,52] and **3** [50–51], and acetylsulfur chloride [53] were prepared according to literature procedures. Column chromatography: silica gel 60, mesh 70–230. TLC: silica gel coated plates. ^1^H and ^13^C NMR spectra: CD_2_Cl_2_, CDCl_3_, and [*D*_6_]-acetone (locked to Me_4_Si) [60]. The assignments of quaternary C, CH, CH_2_, and CH_3_ were made by using DEPT spectra. Elemental analyses were carried out in the Microanalytical Laboratories of the Organisch-Chemisches Institut, Ruprecht-Karls-Universität Heidelberg, Germany.

### Electrochemistry

Cyclic voltammetry experiments (EG & G potentiostatic instrumentation) were performed under an argon atmosphere in dry and degassed CH_2_Cl_2_ at room temperature and at scan rates of 100, 250, 500, and 1000 mV/s. The electrolyte was Bu_4_NPF_6_ (0.025 M). The working electrode was a 1 mm platinum disk, the counter-electrode was a platinum wire, and the reference electrode was an Ag/AgCl electrode. The potentials were corrected to the internal standard of Fc/Fc^+^ in CH_2_Cl_2_ (*E*_0_^0/+1^ = 450 mV) [61].

### 7-Bromo-10,10′,10″,10′″-tetrahexyl-10*H*,10′*H*,10″*H*,10′″*H*-3,3′:7′,3″:7″,3′″-quaterphenothiazine (**1e**)

1.90 g (2.95 mmol) 7-Bromo-10,10′-dihexyl-10*H*,10′*H*-3,3′-biphenothiazine (**1c**) [32,52], 2.37 g (4.43 mmol) 10-hexyl-3,7-bis-(4,4,5,5-tetramethyl-[1,3,2]-dioxaborolan-2-yl)-10*H*-phenothiazine [50–51], and 2.45 g (17.7 mmol) potassium bicarbonate were dissolved in 100 mL of DME and 20 mL of water. The mixture was degassed by purging with argon gas for 20 min. After the addition of 136 mg of tetrakis(triphenylphosphan)palladium (118 μmol, 4 mol %), the reaction mixture was stirred for 12 h at 85 °C. After cooling to room temperature, 50 mg of Na_2_SO_3_ was added and the reaction mixture stirred for 14 h at room temperature. Then, 3.17 g (6.49 mmol) of 3-bromo-10-hexyl-7-iodo-10*H*-phenothiazine [51] was added and the mixture stirred for 4 d at 85 °C. After the addition of 100 mL of water, the crude product was extracted several times with dichloromethane. The combined organic phases were dried with magnesium sulfate and the solvents removed in vacuo. The residue was chromatographed on silica gel (hexane/acetone 50:1) to give 1.53 g (36%) of **1e** as a yellow resin. ^1^H NMR (300 MHz, CD_2_Cl_2_): *δ* = 0.76–0.81 (m, 12H), 1.18–1.26 (m, 16H), 1.30–1.40 (m, 8H), 1.63–1.75 (m, 8H), 3.69–3.77 (m, 8H), 6.61–6.64 (m, 2H), 6.78–6.84 (m, 7H), 7.02–7.10 (m, 3H), 7.14–7.31 (m, 13H). ^13^C NMR (75 MHz, CD_2_Cl_2_): *δ* = 14.2 (CH_3_), 23.0 (CH_2_), 26.9 (CH_2_), 27.0 (CH_2_), 31.8 (CH_2_), 31.8 (CH_2_), 47.9 (CH_2_), 114.5 (C_quat_), 115.9 (CH), 116.0 (CH), 116.9 (CH), 122.7 (CH), 124.6 (CH), 125.2 (CH), 125.4 (CH), 125.5 (CH), 125.7 (CH), 126.9 (C_quat_), 127.6 (CH), 129.7 (CH), 130.2 (C_quat_), 134.6 (C_quat_), 144.7 (C_quat_). MS (MALDI-TOF) *m*/*z* (%): 1206.3 (M^+^, 100), 1126.4 (M^+^ − Br), 1121.2 (M^+^ − C_6_H_13_, 6), 1041.2 (M^+^ − Br − C_6_H_13_, 6). MS (FAB^+^) *m*/*z* (%): 1206.2 (M^+^, 100), 1121.1 (M^+^ − C_6_H_13_, 45), 1037.0 (M^+^ − 2C_6_H_13_, 10), 951.0 (M^+^ − 3C_6_H_13_, 9), 865.9 (M^+^ − 4C_6_H_13_, 23). IR (KBr): *ν* = 2953, 2927, 2868, 2854, 1604, 1457, 1415, 1378, 1332, 1295, 1252, 1240, 1193, 1147, 806, 746 cm^−1^. UV–vis (CH_2_Cl_2_): *λ*_max_ (*ε*) = 238 (6700), 282 (10500), 326 (3500), 376 nm (4300). Anal. Calcd for C_72_H_77_BrN_4_S_4_ (1206.6): C, 71.67; H, 6.43; N, 4.64; Br, 6.62; S, 10.63. Found: C, 71.54; H, 6.53; N, 4.64; Br, 6.91; S, 10.58.

### Thioacetic acid *S*-(10-hexyl-10*H*-phenothiazin-3-yl) ester (**2a**)

To a cooled solution of 500 mg (1.38 mmol) of 3-bromo-10*H*-hexylphenothiazine (**1a**) in 25 mL of dry THF, 0.55 mL (1.52 mmol, 1.1 equiv) of 2.5 M *n*-butyllithium in hexanes was added dropwise over 5 min at −78 °C (dry ice/acetone). After stirring for 5 min at −78 °C, 49 mg (1.52 mmol, 1.1 equiv) of sulfur was added to the reaction mixture. After stirring for a further 5 min at −78 °C, 0.11 mL (1.52 mmol, 1.1 equiv) of acetyl chloride was added dropwise over 5 min. The solution was allowed to come to room temperature and stirred overnight. Then, 50 mL of water was added and the aqueous phase was extracted several times with small portions of dichloromethane. The combined organic phases were dried with magnesium sulfate and the solvents removed in vacuo. The residue was chromatographed on silica gel (hexane/acetone 10:1) to give 279 mg (57%) of **2a** as a yellow oil. *R_f_* (hexane/acetone 5:1) = 0.45. ^1^H NMR (*D*_6_-acetone, 300 MHz): *δ* = 0.88 (t, ^3^*J* = 6.9 Hz, 3H), 1.28 (m, 4H), 1.46 (m, 2H), 1.77 (m, 2H), 2.35 (s, 3H), 3.97 (t, ^3^*J* = 7.2 Hz, 2H), 6.94 (m, 1H), 7.08 (m, 2H), 7.13 (m, 2H), 7.24 (m, 2H). ^13^C NMR (*D*_6_-acetone, 75 MHz): *δ* = 14.2 (CH_3_), 23.2 (CH_2_), 27.1 (CH_2_), 27.4 (CH_2_), 29.9 (CH_3_), 32.1 (CH_2_), 47.9 (CH_2_), 116.9 (CH), 116.9 (CH), 121.9 (C_quat_), 123.8 (CH), 126.2 (C_quat_), 126.7 (C_quat_), 128.1 (CH), 128.5 (CH), 133.7 (CH), 134.7 (CH), 145.6 (C_quat_), 147.5 (C_quat_), 194.2 (C_quat_). MS (FAB^+^) *m*/*z* (%): 357.3 (M^+^, 100), 314.3 (M^+^ − COCH_3_, 18). IR (film): *ν* = 3061, 2954, 2928, 2855, 1708, 1593, 1486, 1462, 1393, 1377, 1126, 878, 812, 749, 615 cm^−1^. UV–vis (CH_2_Cl_2_): *λ*_max_ (*ε*) = 240 (11600), 266 (24800), 310 nm (6200). Anal. Calcd for C_20_H_23_NOS_2_ (357.1): C, 67.19; H, 6.48; N, 3.92. Found: C, 67.16; H, 6.52; N, 3.86.

### Thioacetic acid *S*-(10-methyl-10*H*-phenothiazin-3-yl) ester (**2b**)

To a cooled solution of 292 mg (1.00 mmol) of 3-bromo-10*H*-methylphenothiazine (**1b**) in 10 mL of dry THF, 0.7 mL (1.1 mmol, 1.1 equiv) of 1.58 M *n*-butyllithium in hexanes was added dropwise over 5 min at −78 °C (dry ice/acetone). After stirring for 5 min at −78 °C, 35 mg (1.1 mmol, 1.1 equiv) of sulfur was added to the reaction mixture. After stirring for a further 5 min at −78 °C, 0.07 mL (1.1 mmol, 1.1 equiv) of acetyl chloride was added dropwise over 5 min. The solution was allowed to come to room temperature and stirred overnight. Then, 50 mL of water was added and the aqueous phase extracted several times with small portions of dichloromethane. The combined organic phases were dried with magnesium sulfate and the solvents removed in vacuo. The residue was chromatographed on silica gel (hexane/acetone 50:1) to give 141 mg (49%) of **2b** as a yellow oil. *R_f_* (hexane/acetone 5:1) = 0.29. ^1^H NMR (CD_2_Cl_2_, 300 MHz): *δ* = 2.38 (s, 3H), 3.36 (s, 3H), 6.84 (m, 2H), 6.96 (dt, ^d^*J* = 1.2 Hz, ^t^*J* = 4.5 Hz, 1H), 7.13 (m, 2H), 7.20 (m, 2H). ^13^C NMR (CD_2_Cl_2_, 75 MHz): *δ* = 30.2 (CH_3_), 35.7 (CH_3_), 114.7 (CH), 114.8 (CH), 121.2 (C_quat_), 122.9 (C_quat_), 123.1 (CH), 124.5 (C_quat_), 127.4 (CH), 127.9 (CH), 133.1 (CH), 134.4 (CH), 145.6 (C_quat_), 147.4 (C_quat_), 194.8 (C_quat_). MS (EI^+^) *m*/*z* (%): 287.0 (M^+^, 100), 245.0 (M^+^ − COCH_3_, 62), 230.3 (M^+^ − COCH_3_ − CH_3_, 68), 212.0 (M^+^ − SCOCH_3_, 16). HR-MS (EI^+^) *m*/*z* Calcd for C_15_H_13_NOS_2_: 287.0438. Found: 287.0458. IR (KBr): *ν* = 3057, 2965, 2883, 2819, 1598 cm^−1^. UV–vis (CH_2_Cl_2_): *λ*_max_ (*ε*) = 264 (71200), 316 nm (12800). Anal. Calcd for C_15_H_13_NOS_2_ (287.0): C, 62.69; H, 4.56; N, 4.87. Found: C, 62.68; H, 4.78; N, 4.81.

### *S*-(10,10′-Dihexyl-10*H*,10′*H*-3,3′-biphenothiazin-7-yl) ethanethioate (**2c**)

To a cooled solution of 500 mg (0.78 mmol) of 7-bromo-10,10′-dihexyl-10*H*,10′*H*-3,3′-biphenothiazine (**1c**) in 25 mL of dry THF, 0.91 mL (1.55 mmol, 2.0 equiv) of 1.7 M *t*-butyllithium in pentane was added dropwise over 5 min at −78 °C (dry ice/acetone). After stirring for 10 min at −78 °C, 95 mg (0.78 mmol, 1.0 equiv) of acetylsulfur chloride [25] was added to the reaction mixture. The solution was allowed to come to room temperature and stirred overnight. Then 30 mL of water was added and the aqueous phase was extracted several times with small portions of dichloromethane. The combined organic phases were dried with magnesium sulfate and the solvents were removed in vacuo. The residue was chromatographed on silica gel (hexane/acetone 50:1) to give 76 mg (15%) of **2c** as a yellow resin. *R_f_* (hexane/acetone 5:1) = 0.42. ^1^H NMR (CD_2_Cl_2_, 300 MHz): *δ* = 0.87 (t, ^3^*J* = 6.6 Hz, 6H), 1.31 (m, 8H), 1.43 (m, 4H), 1.79 (m, 4H), 2.36 (s, 3H), 3.85 (t, ^3^*J* = 6.9 Hz, 4H), 6.86 (m, 1H), 6.90 (m, 4H), 7.14 (m, 3H), 7.29 (m, 4H), 7.31 (m, 1H). ^13^C NMR (CD_2_Cl_2_, 75 MHz): *δ* = 14.2 (CH_3_), 23.0 (CH_2_), 27.1 (CH_2_), 27.1 (CH_2_), 30.1 (CH_3_), 31.9 (CH_2_), 48.1 (CH_2_), 115.8 (CH), 116.0 (CH), 116.2 (CH), 121.2 (C_quat_), 122.7 (CH), 124.7 (C_quat_), 124.9 (C_quat_), 125.3 (C_quat_), 125.3 (CH), 125.4 (C_quat_), 125.6 (CH), 127.7 (CH), 133.4 (CH), 134.2 (CH), 134.4 (C_quat_), 135.1 (C_quat_), 143.9 (C_quat_), 144.7 (C_quat_), 146.9 (C_quat_), 194.9 (C_quat_). MS (FAB^+^) *m*/*z* (%): 638.6 (M^+^, 100), 595.5 (M^+^ − COCH_3_, 12), 553.4 (M^+^ − C_6_H_13_, 14). HR-MS (FAB^+^) *m*/*z* Calcd for C_38_H_42_N_2_OS_3_: 638.2459. Found: 683.2445. IR (film): *ν* = 2960, 2927, 2854, 1706, 1601, 1575, 1459, 1416, 1393, 1377, 1334, 1040, 876, 809, 745, 616 cm^−1^. UV–vis (CH_2_Cl_2_): *λ*_max_ (*ε*) = 276 nm (44900), 324 (18900), 366 nm (15400).

### *S*-[10,10′-Dihexyl-7′-(10-hexyl-10*H*-phenothiazin-3-yl)-10*H*,10′*H*-3,3′-biphenothiazin-7-yl] ethanethioate (**2d**)

To a cooled solution of 800 mg (0.87 mmol) of 7-bromo-10,10′-dihexyl-7′-(10-hexyl-10*H*-phenothiazin-3-yl)-10*H*,10′*H*-3,3′-biphenothiazine (**1d**) in 25 mL of dry THF, 1.07 mL (1.82 mmol, 2.1 equiv) of 1.7 M *t*-butyllithium in pentane was added dropwise over 5 min at °C (dry ice/acetone). After stirring for 10 min at −78 °C, 29 mg (0.91 mmol, 1.1 equiv) of sulfur was added to the reaction mixture. After stirring for a further 10 min at −78 °C, 0.07 mL (0.95 mmol, 1.1 equiv) of acetyl chloride was added dropwise over 5 min. The solution was allowed to come to room temperature and stirred overnight. Then, 50 mL of water was added and the aqueous phase extracted several times with small portions of dichloromethane. The combined organic phases were dried with magnesium sulfate and the solvents removed in vacuo. The residue was chromatographed on silica gel (hexane to hexane/acetone 10:1) to give 536 mg (67%) of **2d** as a yellow resin. *R_f_* (hexane/acetone 5:1) = 0.35. ^1^H NMR (CD_2_Cl_2_, 300 MHz): *δ* = 0.88 (t, ^3^*J* = 3 Hz, 9H), 1.31 (m, 12H), 1.44 (m, 6H), 1.80 (m, 6H), 2.36 (s, 3H), 3.85 (t, ^3^*J* = 6 Hz, 6H), 6.90 (m, 7H), 7.14 (m, 5H), 7.29 (m, 7H). ^13^C NMR (CD_2_Cl_2_, 75 MHz): *δ* = 14.2 (CH_3_), 23.0 (CH_2_), 27.0 (CH_2_), 27.1 (CH_2_), 30.2 (CH_3_), 31.9 (CH_2_), 36.3 (CH_2_), 48.0 (CH_2_), 115.8 (C_quat_), 116.0 (CH), 116.2 (CH), 121.1 (C_quat_), 122.7 (CH), 124.8 (C_quat_), 125.2 (CH), 125.6 (CH), 127.7 (CH), 133.4 (CH), 134.2 (CH), 143.9 (C_quat_), 144.7 (C_quat_), 146.8 (C_quat_), 194.9 (C_quat_). MS (MALDI-TOF) *m*/*z* (%): 919.4 (M^+^, 100), 877.4 (M^+^ − COCH_3_, 4). IR (KBr): *ν* = 2954, 2928, 2855, 1700, 1635, 1458, 1416, 1379, 1241, 1193, 873, 807, 747 cm^−1^. UV–vis (CH_2_Cl_2_): *λ*_max_ (*ε*) = 280 (101000), 326 (38100), 364 nm (31400). Anal. calcd. for C_56_H_61_N_3_OS_4_: C, 73.08; H, 6.68; N, 4.57; S, 13.94. Found: C, 73.08; H, 6.60; N, 4.69; S, 13.99.

### *S*-[10,10′,10″-Trihexyl-7′-(10-hexyl-10*H*-phenothiazin-3-yl)-10*H*,10′*H*,10″*H*-3,3′:7′,3″-terphenothiazin-7-yl] ethanethioate (**2e**)

To a cooled solution of 200 mg (0.17 mmol) of 7-(7′-bromo-10,10′-dihexyl-10*H*,10′*H*-3,3′-biphenothiazin-7-yl)-10,10′-dihexyl-10*H*,10′*H*-3,3′-biphenothiazine (**1e**) in 10 mL of dry THF, 0.20 mL (0.35 mmol, 2.1 equiv) of 1.7 M *t*-butyllithium in pentane was added dropwise over 5 min at −78 °C (dry ice/acetone). After stirring for 10 min at −78 °C, 6.0 mg (0.17 mmol, 1.0 equiv) of sulfur was added to the reaction mixture. After stirring for a further 10 min at −78 °C, 0.013 mL (0.18 mmol, 1.1 equiv) of acetyl chloride was added dropwise over 5 min. The solution was allowed to come to room temperature and stirred overnight. Then, 20 mL of water was added and the aqueous phase extracted several times with small portions of dichloromethane. The combined organic phases were dried with magnesium sulfate and the solvents removed in vacuo. The residue was chromatographed on silica gel (hexane to hexane/acetone 50:1) to give 132 mg (66%) of **2e** as a yellow resin. *R_f_* (hexane/acetone 5:1) = 0.24. ^1^H NMR (CD_2_Cl_2_, 300 MHz): *δ* = 0.87 (m, 12H), 1.31 (m, 16H), 1.43 (m, 8H), 1.79 (m, 8H), 2.36 (s, 3H), 3.86 (m, 8H), 6.89 (m, 8H), 7.12 (m, 7H), 7.31 (m, 10H). ^13^C NMR (CD_2_Cl_2_, 75 MHz): *δ* = 14.2 (CH_3_), 23.0 (CH_2_), 27.0 (CH_2_), 30.2 (CH_3_), 31.9 (CH_2_), 48.3 (CH_2_), 115.9 (CH), 121.8 (C_quat_), 125.5 (C_quat_), 127.8 (C_quat_), 131.1 (CH), 132.8 (CH), 133.5 (CH), 134.3 (CH), 140.1 (C_quat_), 140.8 (C_quat_), 146.3 (C_quat_), 148.1 (C_quat_), 194.9 (C_quat_). MS (MALDI-TOF) *m*/*z*: 1200.5 (M^+^), 1158.5 (M^+^ − COCH_3_), 1126.5 (M^+^ − SCOCH_3_), 1116.4 (M^+^ − C_6_H_13_). IR (KBr): *ν* = 2955, 2928, 2854, 1706, 1634, 1604, 1575, 1459, 1415, 1379, 1333, 1241, 1192, 1106, 1062, 874, 807, 746, 616 cm^−1^. UV/vis (CH_2_Cl_2_): *λ*_max_ (*ε*) = 282 (92900), 326 (33800), 362 nm (31000).

### *S*,*S*′-(10-Hexyl-10*H*-phenothiazine-3,7-diyl) bis(ethanethioate) (**4**)

To a cooled solution of 800 mg (1.82 mmol) of 3,7-dibromo-10*H*-hexylphenothiazine (**3**) in 25 mL of dry THF, 4.38 mL (7.44 mmol, 4.1 equiv) of 1.7 M *t*-butyllithium in pentane was added dropwise over 5 min at −78 °C (dry ice/acetone). After stirring for 5 min at −78 °C, 122 mg (3.81 mmol, 2.1 equiv) sulfur was added to the reaction mixture. After stirring for a further 10 min at −78 °C, 0.27 mL (3.72 mmol, 2.1 equiv) of acetyl chloride was added dropwise over 5 min. The solution was allowed to come to room temperature and stirred overnight. Then, 50 mL of water was added and the aqueous phase extracted several times with small portions of dichloromethane. The combined organic phases were dried with magnesium sulfate and the solvents removed in vacuo. The residue was chromatographed on silica gel (hexane to hexane/acetone 50:1) to give 317 mg (41%) of **4** as a yellow oil. *R_f_* (hexane/acetone 5:1) = 0.18. ^1^H NMR (CD_2_Cl_2_, 300 MHz): *δ* = 0.91 (t, ^3^*J* = 6.9 Hz, 3H), 1.33 (m, 4H), 1.42 (m, 2H), 1.80 (m, 2H), 2.38 (s, 6H), 3.85 (t, ^3^*J* = 7.2 Hz, 2H), 6.88 (m, 1H), 6.90 (m, 1H), 7.12 (m, 2H), 7.17 (m, 1H), 7.20 (m, 1H). ^13^C NMR (CD_2_Cl_2_, 75 MHz): *δ* = 14.2 (CH_3_), 23.0 (CH_2_), 26.9 (CH_2_), 27.0 (CH_2_), 30.2 (CH_3_), 31.8 (CH_2_), 48.2 (CH_2_), 116.3 (CH), 121.8 (C_quat_), 125.2 (C_quat_), 133.5 (CH), 134.3 (CH), 146.3 (C_quat_), 194.7 (C_quat_). MS (MALDI-TOF) *m*/*z*: 431.0 (M^+^), 388 (M^+^ − COCH_3_), 356 (M^+^ − SCOCH_3_). IR (film): *ν* = 2955, 2927, 2858, 1707, 1590, 1564, 1463, 1393, 1352, 1332, 1265, 1250, 1124, 949, 813, 615 cm^−1^. UV–vis (CH_2_Cl_2_): *λ*_max_ (*ε*) = 272 (36000), 326 nm (7100). Anal. Calcd for C_20_H_25_NO_2_S_3_ (431.1): C, 61.22; H, 5.84; N, 3.24; S, 22.29. Found: C, 61.27; H, 5.95; N, 3.25; S, 20.70.

### SAM preparation and ellipsometry

The (oligo)phenothiazinyl thioacetates **2a**, **2c–e**, and **4** were dissolved under an argon atmosphere in dry THF to give a 10^−4^ M solutions. Au-coated silicon wafers (surface area: 1 cm^2^) were placed in these solutions. Upon the addition of a few drops of a concentrated solution of aqueous ammonia the thioacetates were saponified to liberate the thiol functionality necessary for chemisorption and SAM formation on gold. After 24 h the wafers were removed from the solution and rinsed several times with dry THF.

The thickness of the formed organic layers was determined by means of spectral ellipsometry (M-44, J.A. Woollam, USA) applying a 3-layer model consisting of gold substrate, organic layer, and ambient [62]. The organic layer was described by means of a Cauchy model, with the first two Cauchy parameters chosen such to yield a refractive index of 1.490 at 500 nm, which resulted from a study on biphenylthiolates on gold in excellent agreement with theory [63].

## Supporting Information

File 1 Molecular modeling coordinates of **2a**, **2c**, **2d**, **2e**, and **4**, cyclic voltammograms of **2a**, **2b**, **2c**, **2d**, **2e**, and **4**, and absorption and emission spectra of **2d** and **2e**.
