# Supplementary material for: Synthesis, electronic properties and self-assembly on Au{111} of thiolated (oligo)phenothiazines
Source: Beilstein J Org Chem. 2010 Jul 2;6:72. doi: 10.3762/bjoc.6.72 (PMC2919263; doi:10.3762/bjoc.6.72)
Supplement: File 1 — Molecular modeling coordinates of 2a, 2c, 2d, 2e, and 4, cyclic voltammograms of 2a, 2b, 2c, 2d, 2e, and 4, and absorption and emission spectra of 2d and 2e. [file Beilstein_J_Org_Chem-06-72-s001.pdf]

Synthesis, Electronic Properties and Self-Assembly on Au{111} of Thiolated (Oligo)phenothiazines

Adam W. Franz,[a] *Svetlana Stoycheva,*[b] *Michael Himmelhaus,*[b] *and Thomas J. J. Müller**[a]

[a] Institut für Organische Chemie und Makromolekulare Chemie, Heinrich-Heine-Universität Düsseldorf, Universitätsstr. 1, D-40225 Düsseldorf, Germany.

[b] Physikalisch-Chemisches Institut, Ruprecht-Karls-Universität Heidelberg, Im Neuenheimer Feld 253, D-69120 Heidelberg, Germany.

ThomasJJ.Mueller@uni-duesseldorf.de

Supporting Information

1) Molecular modeling coordinates of 2a, 2c, 2d, 2e, and 4 **3**

Molecular modeling coordinates (B3LYP/3-21 G) of **2a** 3

Molecular modeling coordinates (B3LYP/3-21 G) of **2c** 4

Molecular modeling coordinates (B3LYP/3-21 G) of **2d** 5

Molecular modeling coordinates (B3LYP/3-21 G) of **2e** 7

Molecular modeling coordinates (B3LYP/3-21 G) of **4** 9

2) Cyclic voltammograms of 2a, 2b, 2c, 2d, 2e, and 4 **10**
Cyclic voltammogram of **2a** 10
Cyclic voltammogram of **2b** 10
Cyclic voltammogram of **2c** 11
Cyclic voltammogram of **2d** 11
Cyclic voltammogram of **2e** 12
Cyclic voltammogram of **4** 12

3) Absorption and emission spectra of 2d, and 2e **13**
Absorption and emission spectra of **2d** 13
Absorption and emission spectra of **2e** 13

4) References **14**

1) Molecular modeling coordinates of **2a, 2c, 2d, 2e** and **4** (the hexyl groups were replaced by methyl groups). The DFT calculations were carried out using the Gaussian software package.[1]

Table 1: Atom coordinates and total energies (in Hartrees) of **2a**, according to DFT B3LYP/3-21G calculations.

Center Number	Atomic Number	Atomic Type	Coordinates (Angstroms) X	Y	Z
1	6	0	4.005448	0.461579	-0.866805
2	6	0	2.835283	0.282539	-0.110389
3	6	0	2.534772	-1.015195	0.332434
4	6	0	3.350588	-2.095176	0.020190
5	6	0	4.524115	-1.898594	-0.712182
6	6	0	4.847868	-0.614858	-1.148385
7	1	0	4.253448	1.442584	-1.248942
8	1	0	3.073825	-3.084568	0.364055
9	1	0	5.169629	-2.738142	-0.938322
10	1	0	5.749679	-0.446158	-1.725051
11	6	0	-1.677130	2.016726	-0.279130
12	1	0	-2.326392	2.795823	-0.658505
13	6	0	-2.224439	0.829138	0.200121
14	6	0	-0.011763	0.012041	0.671478
15	6	0	0.568880	1.198083	0.192558
16	6	0	-0.294937	2.195861	-0.290467
17	1	0	0.112122	3.110813	-0.698798
18	6	0	-1.385445	-0.186194	0.663223
19	1	0	-1.793440	-1.123329	1.010915
20	7	0	1.973234	1.368909	0.188093
21	16	0	-4.056292	0.665095	0.243377
22	16	0	1.076537	-1.264242	1.422730
23	6	0	2.513751	2.736537	0.103210
24	1	0	2.535575	3.132013	-0.922781
25	1	0	1.899216	3.393725	0.723073
26	1	0	3.530500	2.734359	0.502945
27	6	0	-4.300282	-1.066084	-0.547380
28	8	0	-3.384353	-1.839607	-0.746862
29	6	0	-5.768447	-1.278135	-0.837263
30	1	0	-6.380967	-0.901237	-0.013611
31	1	0	-6.036951	-0.722628	-1.743631
32	1	0	-5.957557	-2.342003	-0.998086

HF= -1497.9606486

Table 2: Atom coordinates and total energies (in Hartrees) of **2c**, according to DFT B3LYP/3-21G calculations.

Center Number	Atomic Number	Atomic Type	Coordinates (Angstroms) X	Y	Z
1	6	0	-8.301158	-0.769291	0.494944
2	6	0	-7.036989	-0.709635	-0.115673
3	6	0	-6.315020	-1.907407	-0.240551
4	6	0	-6.817751	-3.111000	0.236730
5	6	0	-8.086875	-3.159458	0.819077
6	6	0	-8.825381	-1.983492	0.940453
7	1	0	-8.873721	0.137672	0.634896
8	1	0	-6.221864	-4.010361	0.137165
9	1	0	-8.486017	-4.101739	1.173290
10	1	0	-9.807607	-2.001302	1.397860
11	6	0	-5.115386	0.795392	-0.450696
12	6	0	-4.154822	-0.217355	-0.606804
13	6	0	-3.285654	2.348929	-0.029788
14	1	0	-2.959086	3.350816	0.222652
15	6	0	-2.333782	1.329365	-0.172488
16	6	0	-2.800506	0.032708	-0.451630
17	1	0	-2.094015	-0.776762	-0.591863
18	6	0	-4.648097	2.086113	-0.150732
19	1	0	-5.354685	2.888011	0.015589
20	16	0	-4.710901	-1.887744	-1.137406
21	7	0	-6.496413	0.515823	-0.581102
22	6	0	-7.417838	1.635642	-0.838506
23	1	0	-7.690353	2.191192	0.070628
24	1	0	-6.943897	2.322427	-1.543893
25	1	0	-8.327095	1.240490	-1.297578
26	6	0	-0.883003	1.604200	-0.029555
27	6	0	-0.039694	0.704230	0.645341
28	6	0	-0.304724	2.764792	-0.563342
29	6	0	1.310976	0.974193	0.798188
30	1	0	-0.453518	-0.192187	1.091628
31	6	0	1.060031	3.013907	-0.439501
32	1	0	-0.922182	3.459475	-1.120482
33	6	0	1.900928	2.126180	0.252572
34	1	0	1.478066	3.894543	-0.908233
35	6	0	3.916038	0.055006	0.911053
36	16	0	2.341047	-0.141171	1.836032
37	7	0	3.288422	2.368821	0.388590
38	6	0	3.785327	3.741306	0.190014
39	1	0	3.074521	4.438454	0.639897
40	1	0	4.743459	3.843098	0.705090
41	1	0	3.920781	4.003775	-0.869232
42	6	0	4.220147	1.302540	0.342278
43	6	0	5.470600	1.441642	-0.279874
44	1	0	5.732221	2.375678	-0.758640
45	6	0	4.803877	-1.008086	0.845759
46	1	0	4.527093	-1.964484	1.272700
47	6	0	6.055000	-0.833544	0.248456
48	6	0	6.392804	0.394644	-0.312575
49	1	0	7.346347	0.521172	-0.800604
50	16	0	7.170692	-2.308266	0.295590
51	6	0	8.602666	-1.942857	-0.887794
52	6	0	9.447227	-3.192879	-1.025277
53	1	0	9.027480	-3.815125	-1.825339
54	1	0	10.465002	-2.906417	-1.301557
55	1	0	9.450337	-3.775235	-0.101442
56	8	0	8.802202	-0.876935	-1.447861

HF=-2446.7026242

Table B: Atom coordinates and total energies (in Hartrees) of **2d**, according to DFT B3LYP/3-21G calculations.

Center Number	Atomic Number	Atomic Type	Coordinates (Angstroms) X	Y	Z
1	6	0	-4.160206	-4.128755	0.008498
2	6	0	-2.932991	-3.670437	0.516378
3	6	0	-2.839672	-2.308050	0.844027
4	6	0	-3.906970	-1.441847	0.665720
5	6	0	-5.143016	-1.908193	0.184352
6	6	0	-5.245507	-3.268584	-0.139402
7	1	0	-4.262653	-5.161388	-0.296668
8	1	0	-3.795410	-0.401305	0.946620
9	1	0	-6.172468	-3.650249	-0.550678
10	6	0	-0.505523	-4.099778	0.455970
11	6	0	-0.111072	-2.789633	0.773566
12	6	0	1.771124	-4.510143	-0.310616
13	1	0	2.491772	-5.183980	-0.758711
14	6	0	2.155090	-3.198063	0.000984
15	6	0	1.177854	-2.339374	0.534211
16	1	0	1.442553	-1.324926	0.808046
17	6	0	0.465331	-4.947624	-0.103246
18	1	0	0.194113	-5.951492	-0.401018
19	16	0	-1.302005	-1.687656	1.637900
20	7	0	-1.830066	-4.540782	0.686396
21	6	0	-2.084902	-5.989976	0.757407
22	1	0	-2.192751	-6.459542	-0.231002
23	1	0	-1.255455	-6.464918	1.286651
24	1	0	-3.000602	-6.155538	1.329907
25	6	0	3.546327	-2.730803	-0.215672
26	6	0	3.811382	-1.445873	-0.721194
27	6	0	4.641903	-3.551991	0.086937
28	6	0	5.113385	-1.021553	-0.935921
29	1	0	2.990490	-0.791095	-0.988372
30	6	0	5.949013	-3.108047	-0.095862
31	1	0	4.469769	-4.533087	0.513380
32	6	0	6.216232	-1.832075	-0.619777
33	1	0	6.768136	-3.750701	0.196996
34	6	0	6.982258	1.016988	-0.903102
35	16	0	5.411655	0.596315	-1.758331
36	7	0	7.539867	-1.370615	-0.812397
37	6	0	8.627906	-2.361666	-0.876123
38	1	0	8.278153	-3.228135	-1.442314
39	1	0	9.472518	-1.918245	-1.408650
40	1	0	8.967271	-2.696262	0.114854
41	6	0	7.877739	-0.014840	-0.578276
42	6	0	9.104731	0.351562	-0.003737
43	1	0	9.810233	-0.413098	0.292413
44	6	0	7.284680	2.346721	-0.651283
45	1	0	6.560572	3.115892	-0.891443
46	6	0	8.526574	2.686622	-0.108458
47	6	0	9.441385	1.688357	0.213722
48	1	0	10.387601	1.947664	0.661677
49	16	0	8.831236	4.498666	0.105996
50	6	0	10.383877	4.664959	1.176709
51	6	0	10.574319	6.126388	1.527101
52	1	0	9.985052	6.353764	2.424010
53	1	0	11.629930	6.305546	1.745512
54	1	0	10.234713	6.776609	0.717978
55	8	0	11.102516	3.742828	1.527553
56	6	0	-6.297532	-0.989383	0.030837
57	6	0	-6.123800	0.318402	-0.455484
58	6	0	-7.596194	-1.390030	0.376374
59	6	0	-7.206947	1.169193	-0.609558
60	1	0	-5.139031	0.656793	-0.754949
61	6	0	-8.676700	-0.519946	0.253289

62	1	0	-7.756224	-2.378895	0.789474
63	6	0	-8.507958	0.780710	-0.250313
64	1	0	-9.655346	-0.847811	0.577074
65	6	0	-8.252681	3.725775	-0.464611
66	6	0	-9.436365	3.057862	-0.112039
67	16	0	-6.964070	2.810088	-1.402454
68	7	0	-9.596892	1.674897	-0.382218
69	6	0	-10.960930	1.120519	-0.412879
70	1	0	-11.360023	0.894217	0.586652
71	1	0	-11.619521	1.842506	-0.901435
72	1	0	-10.951551	0.203067	-1.006215
73	6	0	-10.442738	3.802921	0.525368
74	1	0	-11.356109	3.314929	0.837754
75	6	0	-9.086452	5.806753	0.432031
76	1	0	-8.948263	6.861214	0.636229
77	6	0	-10.274693	5.165166	0.777707
78	1	0	-11.071801	5.717363	1.261458
79	6	0	-8.067140	5.072450	-0.179714
80	1	0	-7.131962	5.547268	-0.451413

HF=-3395.4443006

Table 4: Atom coordinates and total energies (in Hartrees) of **2e**, according to DFT B3LYP/3-21G calculations.

Center Number	Atomic Number	Atomic Type	Coordinates (Angstroms) X	Y	Z
1	6	0	1.519779	-6.805646	0.342913
2	6	0	2.334711	-5.776886	0.843834
3	6	0	1.734892	-4.525320	1.058894
4	6	0	0.398566	-4.298940	0.769205
5	6	0	-0.420048	-5.334836	0.285538
6	6	0	0.168084	-6.591688	0.084109
7	1	0	1.949520	-7.774563	0.127167
8	1	0	-0.025165	-3.319890	0.958727
9	1	0	-0.428117	-7.399395	-0.323520
10	6	0	4.657583	-4.961192	0.850434
11	6	0	4.344184	-3.609997	1.069779
12	6	0	6.863005	-4.242359	0.104922
13	1	0	7.834328	-4.500753	-0.299786
14	6	0	6.539331	-2.894100	0.315401
15	6	0	5.250099	-2.598339	0.792869
16	1	0	4.969465	-1.570394	0.989188
17	6	0	5.938533	-5.253879	0.353433
18	1	0	6.207954	-6.278062	0.133060
19	16	0	2.729225	-3.191865	1.843010
20	7	0	3.710170	-5.980605	1.109568
21	6	0	4.194510	-7.360567	1.284955
22	1	0	4.376286	-7.880349	0.333030
23	1	0	5.125101	-7.333397	1.856679
24	1	0	3.451890	-7.920840	1.857992
25	6	0	7.517570	-1.813779	0.039060
26	6	0	7.107849	-0.594716	-0.529686
27	6	0	8.879073	-1.968291	0.338011
28	6	0	8.026610	0.404570	-0.808347
29	1	0	6.068166	-0.445295	-0.795484
30	6	0	9.793731	-0.948606	0.087056
31	1	0	9.220314	-2.880321	0.813185
32	6	0	9.389638	0.261430	-0.501589
33	1	0	10.826916	-1.085987	0.376239
34	6	0	8.643924	3.097396	-0.922895
35	16	0	7.475149	1.913667	-1.703048
36	7	0	10.309924	1.303350	-0.766077
37	6	0	11.743914	0.973377	-0.835427
38	1	0	11.857226	0.017386	-1.352108
39	1	0	12.251445	1.745017	-1.419034
40	1	0	12.220517	0.902964	0.153101
41	6	0	9.938206	2.659867	-0.597471
42	6	0	10.834131	3.612897	-0.088853
43	1	0	11.831024	3.311923	0.203912
44	6	0	8.252637	4.413535	-0.728665
45	1	0	7.238364	4.711737	-0.965112
46	6	0	9.173245	5.348814	-0.248826
47	6	0	10.468886	4.950672	0.067533
48	1	0	11.170388	5.665690	0.467372
49	16	0	8.545933	7.082950	-0.104717
50	6	0	9.818969	8.034832	0.924867
51	6	0	9.260594	9.408805	1.232882
52	1	0	8.656202	9.346418	2.146264
53	1	0	10.089253	10.099622	1.406987
54	1	0	8.623874	9.770337	0.422598
55	8	0	10.903997	7.603975	1.280968
56	6	0	-1.854217	-5.098937	-0.011560
57	6	0	-2.277403	-3.907992	-0.627585
58	6	0	-2.832414	-6.051271	0.307887
59	6	0	-3.613124	-3.700948	-0.933920
60	1	0	-1.548124	-3.157872	-0.909771
61	6	0	-4.178369	-5.823254	0.031729

62	1	0	-2.542704	-6.962878	0.817033
63	6	0	-4.600486	-4.644456	-0.606359
64	1	0	-4.909426	-6.559525	0.337434
65	6	0	-5.769324	-1.975800	-1.178162
66	6	0	-6.516155	-3.106857	-0.812041
67	16	0	-4.093847	-2.210200	-1.896239
68	7	0	-5.963890	-4.407998	-0.901098
69	6	0	-6.882909	-5.558638	-0.938730
70	1	0	-7.234227	-5.865042	0.057210
71	1	0	-7.746799	-5.295815	-1.553753
72	1	0	-6.368005	-6.400787	-1.407076
73	6	0	-7.824763	-2.892712	-0.347244
74	1	0	-8.429467	-3.734251	-0.037321
75	6	0	-7.606941	-0.484694	-0.631519
76	6	0	-8.363892	-1.610526	-0.277310
77	1	0	-9.389977	-1.483247	0.046636
78	6	0	-6.287411	-0.694444	-1.068528
79	1	0	-5.660181	0.152865	-1.317985
80	6	0	-8.176012	0.883439	-0.553500
81	6	0	-7.924824	1.836800	-1.550448
82	6	0	-8.997382	1.262864	0.522716
83	6	0	-8.480126	3.112473	-1.484832
84	1	0	-7.323462	1.564037	-2.409624
85	6	0	-9.518902	2.544405	0.602045
86	1	0	-9.193647	0.561524	1.324871
87	6	0	-9.286139	3.499411	-0.400970
88	1	0	-8.303579	3.804608	-2.297112
89	16	0	-10.449433	3.051790	2.104541
90	7	0	-9.853235	4.793577	-0.320549
91	6	0	-11.613627	4.216387	1.288088
92	6	0	-11.156102	4.984293	0.205026
93	6	0	-12.897682	4.369213	1.794997
94	6	0	-12.037032	5.932802	-0.341633
95	6	0	-13.754027	5.333481	1.257389
96	1	0	-13.218719	3.745047	2.620377
97	6	0	-13.313959	6.115857	0.190899
98	1	0	-11.729746	6.522283	-1.194879
99	1	0	-14.747979	5.465550	1.666448
100	1	0	-13.968146	6.862273	-0.244088
101	6	0	-9.256509	5.872533	-1.126330
102	1	0	-8.169683	5.762742	-1.105723
103	1	0	-9.594969	5.870504	-2.172664
104	1	0	-9.516144	6.831697	-0.672119

HF=-4344.186148

Table 5: Atom coordinates and total energies (in Hartrees) of **4**, according to DFT B3LYP/3-21G calculations.

Center Number	Atomic Number	Atomic Type	Coordinates (Angstroms) X	Y	Z
1	6	0	2.337461	1.706874	0.074467
2	6	0	1.230746	1.089456	-0.529000
3	6	0	1.381879	-0.242905	-0.946840
4	6	0	2.567926	-0.934638	-0.753225
5	6	0	3.664890	-0.286223	-0.179493
6	6	0	3.552513	1.038752	0.231999
7	1	0	2.253998	2.721355	0.440358
8	1	0	2.641849	-1.970282	-1.062064
9	1	0	4.386152	1.529358	0.708860
10	7	0	0.000016	1.768987	-0.700023
11	16	0	0.000022	-1.053329	-1.847571
12	6	0	-1.230716	1.089435	-0.528972
13	6	0	-2.337390	1.706745	0.074692
14	6	0	-1.381810	-0.242943	-0.946763
15	6	0	-3.552388	1.038554	0.232334
16	1	0	-2.253934	2.721191	0.440673
17	6	0	-2.567789	-0.934757	-0.753014
18	6	0	-3.664732	-0.286424	-0.179165
19	1	0	-4.385988	1.529073	0.709345
20	1	0	-2.641660	-1.970410	-1.061842
21	6	0	0.000051	3.241391	-0.659199
22	1	0	-0.886565	3.604605	-1.184182
23	1	0	-0.000005	3.646482	0.363040
24	1	0	0.886770	3.604545	-1.184056
25	16	0	-5.210634	-1.293776	-0.053567
26	16	0	5.210917	-1.293377	-0.054108
27	6	0	-6.341158	-0.390485	1.168922
28	6	0	-7.532951	-1.275285	1.470579
29	1	0	-7.285617	-1.918045	2.324503
30	1	0	-8.385154	-0.646870	1.741106
31	1	0	-7.781575	-1.913073	0.619798
32	6	0	6.340597	-0.390868	1.169833
33	6	0	7.532956	-1.275244	1.470484
34	1	0	8.384496	-0.646643	1.742640
35	1	0	7.285665	-1.919896	2.322987
36	1	0	7.782528	-1.911187	0.618586
37	8	0	6.132653	0.718197	1.634315
38	8	0	-6.133461	0.718802	1.632999

HF=-2045.9902446

2) Cyclic voltammetry of 2a-e and 4

Electrochemistry: Cyclic voltammetry experiments (EG & G potentiostatic instrumentation) were performed under argon in dry and degassed CH_2Cl_2 at room temperature and at scan rates of 100, 250, 500, and 1000 mVs^{-1}. The electrolyte was Bu_4NPF_6 (0.025 M). The working electrode was a 1 mm platinum disk, the counter-electrode was a platinum wire, and the reference electrode was a Ag/AgCl electrode. The potentials were corrected to the internal standard of Fc/Fc^+ in CH_2Cl_2 ($E_0^{0/+1}$= 450 mV).[2]

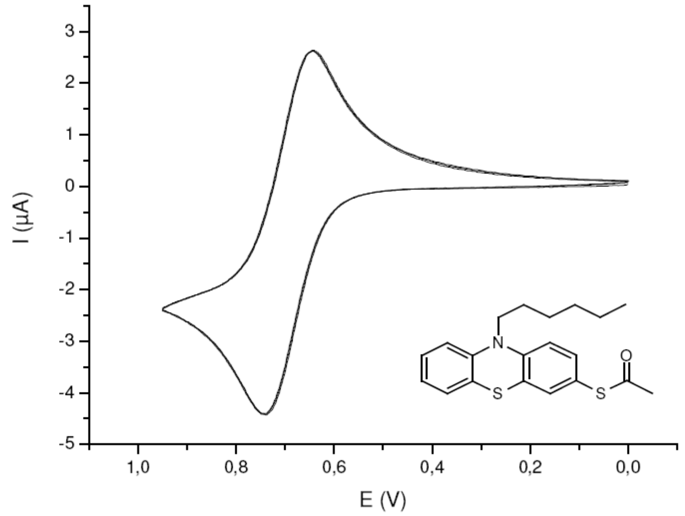

Figure 1. Cyclic voltammogram of thioacetate **2a** (recorded in CH_2Cl_2, T = 293 K; 0.1 *M* electrolyte $[Bu_4N][PF_6]$; ν = 100 mV/s; Pt-working electrode, Ag/AgCl-reference and Pt-counter electrode).

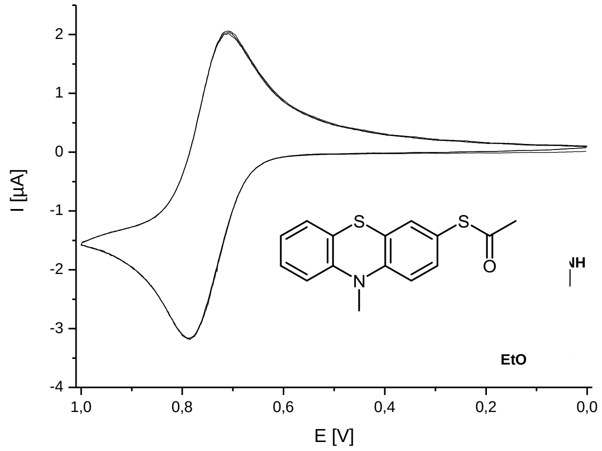

Figure 2. Cyclic voltammogram of thioacetate **2b** (recorded in CH_2Cl_2, T = 293 K; 0.1 *M* electrolyte $[Bu_4N][PF_6]$; ν = 100 mV/s; Pt-working electrode, Ag/AgCl-reference and Pt-counter electrode).

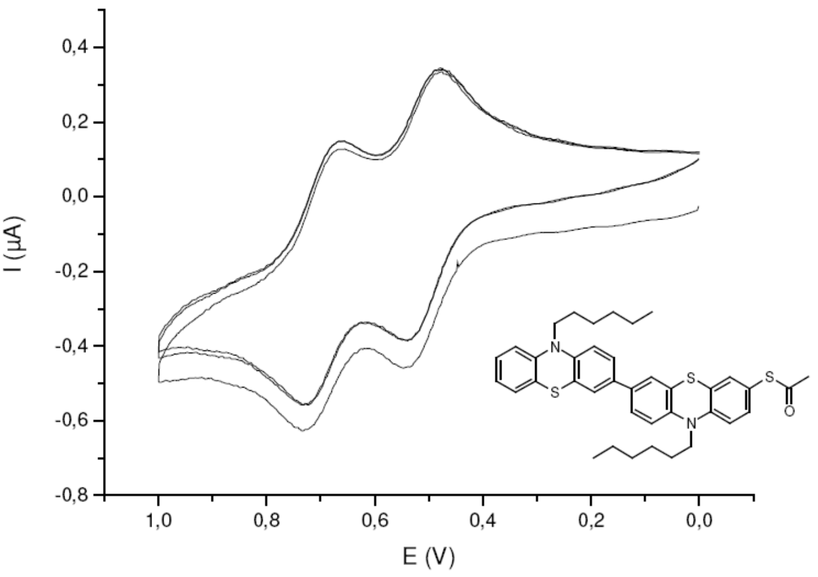

Figure 3. Cyclic voltammogram of thioacetate **2c** (recorded in CH_2Cl_2, T = 293 K; 0.1 *M* electrolyte [Bu_4N][PF_6]; ν = 100 mV/s; Pt-working electrode, Ag/AgCl-reference and Pt-counter electrode).

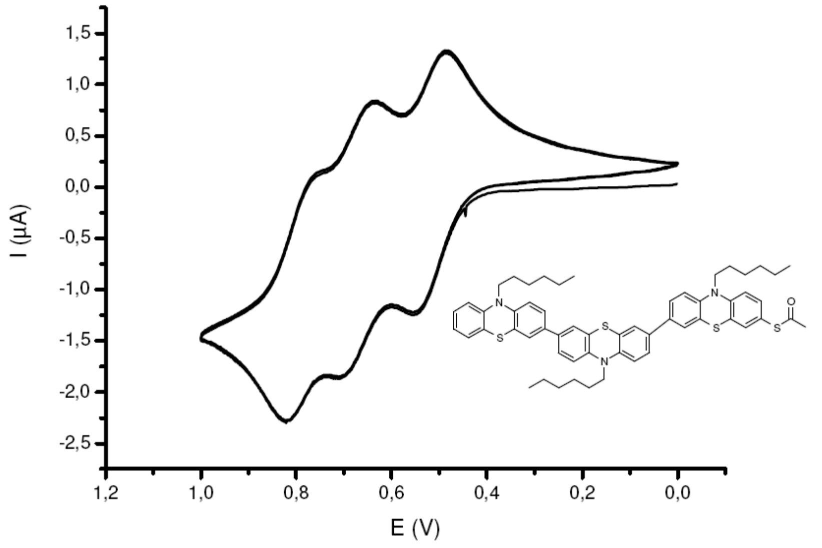

Figure 4. Cyclic voltammogram of thioacetate **2d** (recorded in CH_2Cl_2, T = 293 K; 0.1 *M* electrolyte [Bu_4N][PF_6]; ν = 100 mV/s; Pt-working electrode, Ag/AgCl-reference and Pt-counter electrode).

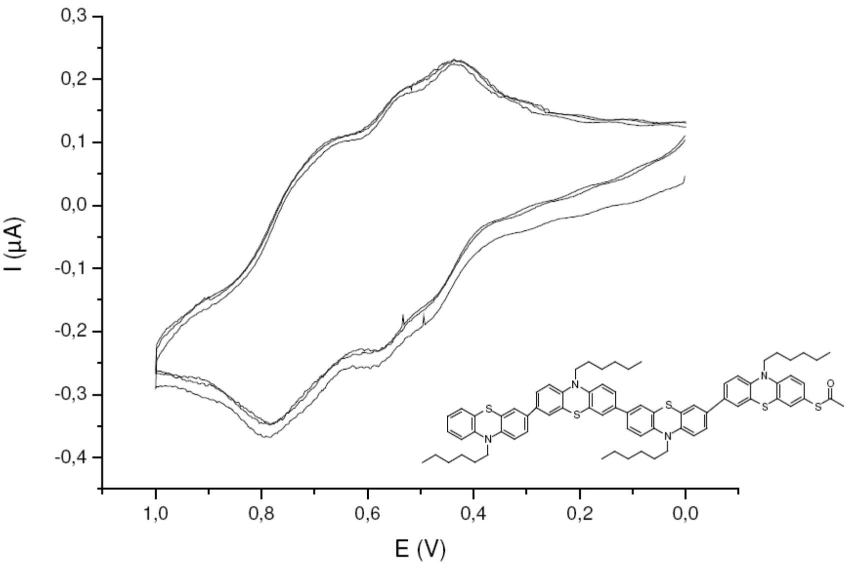

Figure 5. Cyclic voltammogram of thioacetate **2e** (recorded in CH_2Cl_2, T = 293 K; 0.1 *M* electrolyte $[Bu_4N][PF_6]$; ν = 100 mV/s; Pt-working electrode, Ag/AgCl-reference and Pt-counter electrode).

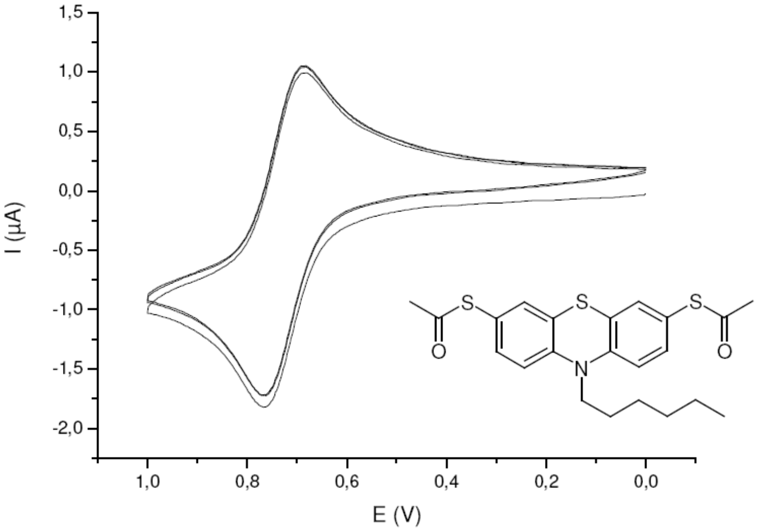

Figure 6. Cyclic voltammogram of thioacetate **4** (recorded in CH_2Cl_2, T = 293 K; 0.1 *M* electrolyte $[Bu_4N][PF_6]$; ν = 100 mV/s; Pt-working electrode, Ag/AgCl-reference and Pt-counter electrode).

3) Absorption and emission spectra of 2d, and 2e

Absorption and emission spectra of **2d**

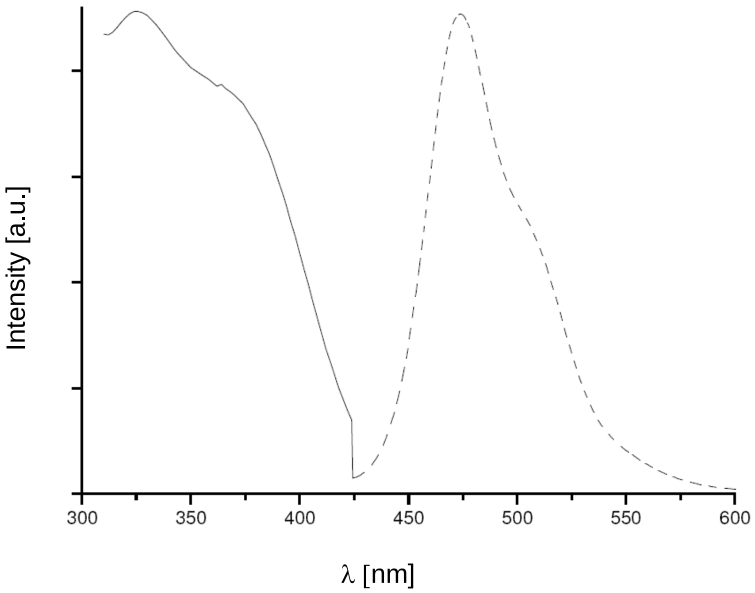

Figure 7. Normalized absorption (solid line) and emission (dashed line) spectra of thioacetate **2d** (recorded in dichloromethane, T = 298 K).

Absorption and emission spectra of **2e**

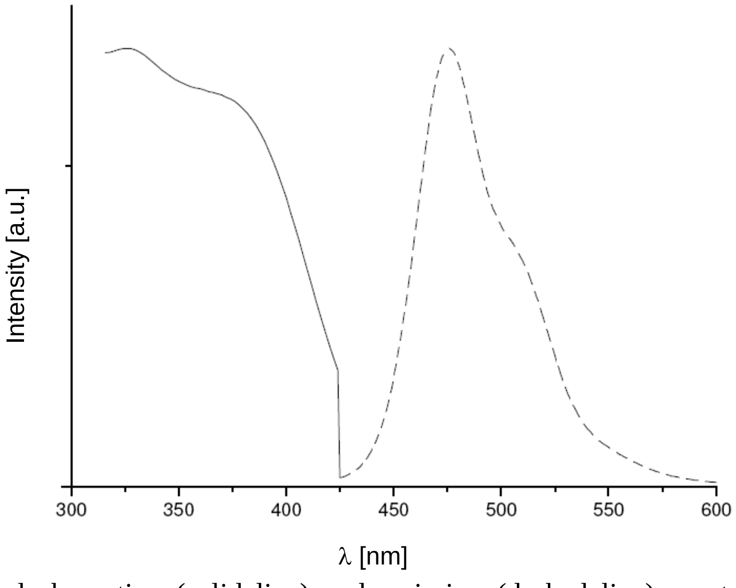

Figure 8. Normalized absorption (solid line) and emission (dashed line) spectra of thioacetate **2e** (recorded in dichloromethane, T = 298 K).

4) References:

(1) Frisch, M. J.; Trucks, G. W:; Schlegel, H. B.; Scuseria, G. E.; Robb, M. A.; Cheeseman, J. R.; Montgomery, J. A. Jr.; Vreven, T.; Kudin, K. N.; Burant, J. C.; Millam, J. M.; Iyengar, S. S.; Tomasi, J.; Barone, V.; Mennucci, B.; Cossi, M.; Scalmani, G.; Rega, N.; Petersson, G. A.; Nakatsuji, H.; Hada, M.; Ehara, M.; Toyota, K.; Fukuda, R.; Hasegawa, J.; Ishida, M.; Nakajima, T.; Honda, Y.; Kitao, O.; Nakai, H.; Klene, M.; Li, X.; Knox, J. E.; Hratchian, H. P.; Cross, J. B.; Adamo, C.; Jaramillo, J.; Gomperts, R.; Stratmann, R. E.; Yazyev, O.; Austin, A. J.; Cammi, R.; Pomelli, C.; Ochterski, J. W.; Ayala, P. Y.; Morokuma, K.; Voth, G. A.; Salvador, P.; Dannenberg, J. J.; Zakrzewski, V. G.; Dapprich, S.; Daniels, A. D.; Strain, M. C.; Farkas, O.; Malick, D. K.; Rabuck, A. D.; Raghavachari, K.; Foresman, J. B.; Ortiz, J. V.; Cui, Q.; Baboul, A. G.; Clifford, S.; Cioslowski, J.; Stefanov, B. B.; Liu, G.; Liashenko, A.; Piskorz, P.; Komaromi, I.; Martin, R. L.; Fox, D. J.; Keith, T.; Al-Laham, M. A.; Peng, C. Y.; Nanayakkara, A.; Challacombe, M.; Gill, P. M.W.; Johnson, B.; Chen, W.; Wong, M. W.; Gonzalez, C.; Pople, J. A.; *Gaussian ´03*, Gaussian, Inc., Pittsburgh PA, **2003**.

(2) Zanello, P. in: *Ferrocenes* (Eds.: Togni, A.; Hayashi, T.), VCH, Weinheim, New York, Basel, Cambridge, Tokyo, **1995**, pp. 317 – 430.
